# Atopic Dermatitis: Identification and Management of Complicating Factors

**DOI:** 10.3390/ijms21082671

**Published:** 2020-04-11

**Authors:** Risa Tamagawa-Mineoka, Norito Katoh

**Affiliations:** Department of Dermatology, Graduate School of Medical Science, Kyoto Prefectural University of Medicine, 465 Kajii-cho, Kawaramachi-Hirokoji, Kamigyo-ku, Kyoto 602-8566, Japan; nkatoh@koto.kpu-m.ac.jp

**Keywords:** atopic dermatitis, complicating factors, aggravating factors, triggering factors, irritants, aeroallergens, food, microbial organisms, contact allergens, sweat, scratching

## Abstract

Atopic dermatitis (AD) is a chronic relapsing inflammatory skin disease, associated with impaired skin barrier function and an atopic background. Various complicating factors, such as irritants, aeroallergens, food, microbial organisms, contact allergens, sweat, and scratching can induce the development of AD symptoms. Irritants, including soap/shampoo and clothes, can cause itching and eczematous lesions. In addition, young children with AD tend to become sensitized to eggs, milk, or peanuts, while older children and adults more often become sensitized to environmental allergens, such as house dust mites (HDM), animal dander, or pollen. Serum-specific IgE levels and skin prick test reactions to food tend to show high negative predictive values and low specificity and positive predictive values for diagnosing food allergy. On the other hand, AD adult patients tend to have severe skin symptoms and exhibit high HDM-specific IgE levels. Microbial organisms, e.g., *Staphylococcus aureus* and *Malassezia furfur*, might contribute to the pathogenetic mechanisms of AD. While sweat plays a major role in maintaining skin homeostasis, it can become an aggravating factor in patients with AD. Furthermore, scratching often exacerbates eczematous lesions. Several patient-specific complicating factors are seen in most cases. The identification and management of complicating factors are important for controlling AD.

## 1. Introduction

Atopic dermatitis (AD) is a common, chronic relapsing inflammatory, multifactorial skin disease, which is characterized by intense pruritus [1,2,3]. It affects up to 20% of children and 1–3% of adults [4]. The mechanisms responsible for the onset and aggravation of AD involve skin barrier dysfunction and an atopic background. In patients with AD, the functions of the intercellular lipids of the stratum corneum are impaired because of abnormal reductions in ceramide levels [5,6]. The horny cell layer, which consists of keratin and filaggrin, is structurally tough. A loss-of-function mutation in filaggrin and filaggrin deficiency related to inflammation have been observed in patients with AD [7,8]. A reduction in skin barrier function might allow stimuli and allergens to penetrate the skin more easily. Interleukin (IL)-33, IL-25, and thymic stromal lymphopoietin (TSLP), which are released from epidermal keratinocytes upon exposure to proteases, allergens, infections, or tissue damage, induce type-2 immune reactions, leading to the induction of allergen-specific IgE antibody production.

With regard to the treatment of AD, topical corticosteroids and topical calcineurin inhibitors are the main treatments for inflammation, whereas the topical application of moisturizers is used to treat cutaneous barrier dysfunction [1,2,9]. Systemic treatment, e.g., oral cyclosporin and UV irradiation, is an option for severe refractory cases [1,2,10]. Several patient-specific complicating factors are seen in most cases. It is important to identify such factors and establish strategies to combat them. This review concisely discusses the identification and management of the complicating factors of AD.

## 2. Irritants

Skin barrier function is impaired in patients with AD. Therefore, there is a tendency for itching and eczematous lesions to develop after AD patients come into contact with irritants (Table 1). To prevent this, AD patients should cleanse their skin gently to get rid of any dirt. Regarding the pH of the corneal layer, a subacidic state is considered healthy. A barrier-dependent increase in pH, e.g., due to the use of neutral-to-alkaline soaps, leads to a reduction in skin barrier function [11]. Soaps and shampoos that contain non-irritant ingredients can be used instead. Furthermore, residual soap and shampoo can induce irritant dermatitis. Therefore, it is important to fully wash away soap/shampoo. Synthetic fabrics and wool also tend to produce itching and irritate the skin [1,2,12,13,14]. A previous study has shown that the irritative capacity of synthetic shirts is significantly higher in patients with AD, while cotton shirts are tolerated best [12]. Thus, AD patients should select non-irritating clothes.

## 3. Aeroallergens

### 3.1. House Dust Mites (HDM)

Aeroallergens, such as HDM, animal dander, and pollen, can lead to the exacerbation of AD. HDM is one of the main allergens blamed for household dust allergies [15]. The most frequently responsible mites are *Dermatophagoides pteronyssinus* (European house dust mite) and *D. farinae* (American house dust mite), which produce various allergens in their bodies and feces. Currently, there are >30 defined mite allergens (Table 2) [16]. It is considered that the most clinically important allergens are Der p 1 and Der p 2 from *D. Pteronyssinus* and Der f 1 and Der f 2 from *D. farinae*. The HDM allergen Der p 1 is found at concentrations of 0.05–0.2 ng/m^3^ in inhalable indoor air [17].

Patients with AD frequently exhibit high total serum IgE levels and environmental allergen-specific IgE. In addition, skin prick testing and atopic patch testing (APT) produce positive reactions to these allergens in AD patients [18,19,20]. APT has been used to test for delayed-type hypersensitivity/eczematous reactions. Langerhans cells from AD patients have been shown to have an increased capacity to present HDM allergen antigens to T cells via the high affinity receptor for IgE (FcεRI) [21,22], suggesting that IgE-mediated delayed-type hypersensitivity reactions to HDM allergens are associated with the pathogenesis of AD [23,24,25]. It has been reported that AD patients that experience delayed-type hypersensitivity reactions tend to have severe skin symptoms and exhibit high total IgE levels and HDM-specific IgE levels [17]. In addition, the positivity rate of patch tests performed with HDM antigens was found to be significantly higher in patients that mainly had eczema lesions on exposed areas, such as the hands, forearms, head, and neck, than in patients who also had eczema lesions on non-exposed areas [26].

Even when a patient’s serum levels of HDM-specific IgE are increased and strong reactions to HDM are seen during skin tests, whether exposure to HDM is considered to be an aggravating factor in that case should be determined based on the patient’s episodes and symptoms [1]. For example, it should be clarified whether the patient develops symptoms of immediate-type hypersensitivity reactions, i.e., wheals, conjunctival or rhinitis symptoms, or asthma attacks, when they are in environments containing large amounts of HDM. Regarding the involvement of delayed-type hypersensitivity reactions in AD, if a patient experiences periods in which their skin symptoms improve or worsen due to changes in their living environment, e.g., due to moving house, travelling, or hospitalization, mites should be considered to be a potential aggravating factor, and the anti-mite measures described below are recommended [1].

Measures that can reduce HDM levels include the use of wood flooring, floor cleaning, vacuum cleaning bedclothes, drying bedclothes in the sun, and the removal of sofas made of cloth and stuffed toys [1,2,14]. While AD lesions were improved by HDM avoidance based on such measures [27,28], reductions in HDM levels had no effect on skin symptoms [29]. The effects of HDM allergen immunotherapy on AD lesions have been investigated by several groups [30,31]. Ridolo et al. reported that the treatment of AD with the causative aeroallergen can be used as an add-on therapy in selected patients who are non-responsive to conventional therapy [30].

### 3.2. Animal Dander

In most modern cities, cats and dogs are often present in houses, and some individuals become allergic to proteins found in cat or dog dander. Cats are the second most common source of indoor environmental allergens after HDM. Cat allergens are found at high levels (20 ng/m^3^) [32]. Ten cat allergens have been identified. The major allergen responsible for symptoms is Fel d 1, a secretoglobin [33].

Čelakovská et al. reported that persistent AD lesions occurred more often in patients that had become sensitized to animal dander or HDM [34]. In addition, IgE sensitization to animal dander or HDM might increase the risk of patients developing asthma or rhinitis [34]. In patients with cat allergies, allergen immunotherapy has been performed with cat dander extract, which was effective at treating cat allergy symptoms, especially respiratory symptoms [35]. In addition, weaker skin test reactions to the cat extract were seen in the treated group than in the placebo group [35].

### 3.3. Pollen

Pollen grains, which represent a small fraction of the viable biological particles present in the air, are important aeroallergens in the outdoor environment. Airborne pollen can exacerbate AD lesions [36,37,38]. In a previous study, pronounced eczema flare-ups occurred on exposed rather than covered areas of skin. Epicutaneous patch testing with pollen allergen extract induced eczematous lesions in patients with AD [36,37]. In grass pollen-exposed subjects, the serum levels of chemokine (C-C motif) ligand 17 (CCL17), CCL22, and IL-4 were significantly increased [30]. The preventative measures that can be used against pollen allergies include brushing pollen off clothes, washing your face when arriving home, wearing glasses and masks, and using air conditioning with pollen filters (Table 3) [1,2,14].

## 4. Food

### 4.1. Blood and Skin Tests

Food allergens might contribute to the pathogenetic mechanisms of AD, especially during infancy. Serum-specific IgE levels and skin prick test results exhibit high negative predictive values (95%) and low specificity and positive predictive values (40–60%) for diagnosing food hypersensitivity [14,39,40,41]. These findings indicate that negative test results are helpful for ruling out food allergies; however, positive results only signify sensitization and need to be assessed in combination with clinical findings. Therefore, the effects of food allergens should be evaluated based on the results of oral food challenges, which should be performed after causative food elimination in addition to obtaining a detailed medical history and evaluating the patient’s serum-specific IgE antibody levels and skin test results [1,2,14].

### 4.2. Allergen-Free Diet for Pregnant/Lactating Women

A systematic review of randomized controlled studies that examined the use of allergen-free diets in pregnant/lactating women has been reported [42]. Dietary restrictions involving allergen elimination in pregnant and lactating women did not prevent the onset of AD in infants. Therefore, dietary restrictions in pregnant and lactating women are not needed to prevent the onset of AD in their children [1].

### 4.3. Percutaneous Sensitization in Food Allergy

Lack et al. performed an epidemiological survey of peanut allergies and detected a significant relationship between peanut allergies and the application of skincare preparations containing peanut oil in children [43]. Based on these findings, the dual-allergen exposure hypothesis, which suggests that oral allergen intake induces immunotolerance, whereas allergen exposure via skin with decreased barrier function induces sensitization, has been proposed [44]. Since the proposal of this hypothesis, allergies caused by percutaneous sensitization, in which sensitization is established by the skin coming into contact with foods, has attracted attention as a mechanism that might be responsible for food allergies.

## 5. Microbial Organisms

### 5.1. Staphylococcus Aureus

*Staphylococcus aureus* is frequently detected in AD lesions. Kong et al. reported that the frequency of *Staphylococcus* sequences, particularly *S. aureus*, increased during disease flare-ups and was correlated with the severity of skin symptoms in children with AD [45]. In addition, Simpson et al. demonstrated that AD patients that had been colonized with *S. aureus* had higher levels of type-2 biomarkers (higher blood eosinophil counts and serum levels of total IgE, CCL17, and periostin) and exhibited greater allergen sensitization than both non-colonized AD patients and non-atopic, non-colonized control individuals [46]. Huang et al. investigated the effects of suppressing *S. aureus* growth with sodium hypochlorite (bleach) baths [47]. The AD patients that received bleach baths displayed significantly greater reductions in disease severity compared with the control subjects.

### 5.2. Malassezia Furfur

The characteristic distribution of AD skin lesions, which often affect the head and neck, implies that an association exists between the exacerbation of AD and cutaneous microflora, such as *Malassezia furfur*. Reactivity to *Malassezia* allergens, which was measured based on serum-specific IgE levels, positive skin prick tests, and positive patch tests, was found to be increased in AD patients with head and neck dermatitis [48,49,50]. It has been reported that oral [51] and topical antifungal drugs [52] are effective against AD. Taken together with the findings of previous studies into *S. aureus* and *M. furfur*, further research is required to fully understand the relationship between the microbial organisms found on the skin and the clinical symptoms of AD [1].

## 6. Contact Allergens

### 6.1. Contact Allergy

Allergic contact dermatitis is a delayed-type hypersensitivity reaction to small environmental chemicals, i.e., haptens or prehaptens, that come into contact with the skin. Contact allergies can cause refractory eczematous lesions in patients with AD. In particular, if an AD patient displays an atypical distribution of eczematous lesions, they might be suffering from a contact allergy. According to previous studies, the frequency of contact allergies in AD patients ranges from 26% to 54% [53]. The most common contact allergens are metals, topical drugs, fragrance, and rubber accelerators [53,54,55] (Table 4). In a previous study, the avoidance of products containing allergenic substances markedly or partially improved eczematous lesions in two-thirds of AD patients that exhibited positive patch test reactions [53]. With regard to nickel and cosmetics, females become sensitized to them more often than males, probably because of their greater use of cosmetics and jewelry, especially among females with pierced ears.

### 6.2. Intrinsic AD

AD can be categorized into the IgE-high, extrinsic type and the IgE-normal, intrinsic type [56]. Extrinsic AD is the classical type, which displays a high prevalence and is associated with elevated IgE levels and skin barrier dysfunction due to decreased filaggrin expression. On the other hand, the incidence of intrinsic AD, which predominantly affects females, is approximately 20%. It has been shown that the percentage of interferon-γ-producing Th1 cells in the peripheral blood is significantly higher in intrinsic AD patients than in extrinsic AD patients [57]. Interestingly, patients with intrinsic AD displayed a higher proportion of positive patch test reactions to metals than those with extrinsic AD, and a metal-free diet partially ameliorated skin symptoms in patients with intrinsic AD, but not those with extrinsic AD [53,58]. Furthermore, Yamaguchi et al. reported that the concentration of nickel in sweat was higher in intrinsic AD patients than in extrinsic AD patients and was inversely correlated with serum IgE levels [58]. These findings suggest that metal allergies are a potential cause of intrinsic AD.

## 7. Sweating

### 7.1. The Function and Composition of Sweat

Sweat includes natural moisturizing factors (e.g., lactate, urea, and electrolytes, free amino acids, and pyrrolidone carboxylic acid), antimicrobial peptides (e.g., dermcidin, β-defensins, and cathelicidin), IgA, sodium bicarbonate, pyruvic acid, proteases, and protease inhibitors, and contributes to skin homeostasis, including temperature regulation, skin moisture regulation, and immune functions [59,60]. Several previous studies have reported that the composition of sweat was changed in patients with AD [61,62,63]. Liebke et al. found that the sodium concentration of sweat was significantly higher in children with AD than in healthy children [61]. Moreover, Sugawara et al. showed that patients with AD had significantly reduced levels of sodium, potassium, lactate, urea, and pyrrolidone carboxylic acid in their sweat than healthy controls [62]. These findings suggest that impaired sweating might reduce the levels of natural moisturizing factors and cause dry skin in patients with AD. It is considered that patients with AD are at higher risk of skin infections and *S. aureus* colonization. Rieg et al. found that patients with AD displayed significantly lower levels of dermcidin-derived antimicrobial peptides in their sweat than healthy controls [63]. Furthermore, the skin bacterial count after physical exercise-induced sweating was lower in the AD patients than in the healthy subjects [63]. Imayama et al. demonstrated that AD patients had lower secretory IgA levels in their sweat than healthy controls [64]. These findings suggest that decreased levels of antimicrobial peptides and IgA in sweat might lead to increased susceptibility to skin infections in patients with AD.

### 7.2. Decreased Sweating in AD Patients

Patients with AD often sweat significantly less than healthy individuals [65,66,67]. Several possible mechanisms have been suggested to be responsible for the decreased sweating seen in patients with AD [59,60]. It has previously been reported that horny plugs or mucopolysaccharides were seen in the openings of the sweat ducts in conditions involving sweat retention [68,69]. With regard to sweat-gland functions, acetylcholine-induced sweating responses were reduced in AD patients compared with those seen in healthy controls, and histamine suppressed acetylcholine-induced sweating via H1 receptor-mediated signaling [70,71]. Furthermore, sweat leakage into the surrounding tissues was observed in patients with AD. The leakage of sweat, as demonstrated by sweat gland-specific dermcidin expression in the dermis around the sweat ducts and glands, was specifically detected in the skin of AD patients [72]. Interestingly, the expression of claudin-3, which acts as a component of the tight junctions between the luminal cells throughout the sweat gland, was significantly reduced in patients with AD compared with that observed in healthy individuals [71]. The reductions in sweating induced via these mechanisms might cause skin dryness and increase patients’ susceptibility to infection, resulting in the exacerbation of the symptoms of AD.

### 7.3. Sweat Allergies

While sweat is important for maintaining homeostasis, it is likely to induce pruritus in patients with AD. Hide et al. reported that intradermal tests with autologous sweat induced positive reactions in many patients with AD [73]. In addition, it has been reported that patients with AD exhibited positive reactions to sweat antigens in a histamine release test [73]. Interestingly, Hiragun et al. found that MGL_1304, a fungal protein derived from *Malassezia globosa*, is a major antigen in human sweat, and recombinant MGL_1304 induced histamine release from basophils in most AD patients [74]. Patients with AD might develop immediate-type hypersensitivity reactions to sweat antigens, leading to exacerbated itching and irritation in response to sweating. These findings suggest that mast cells might react to sweat that leaks from weak sweat ducts and glands in patients with AD.

### 7.4. Measures for Sweating

It is not necessary to avoid sweating because sweating is important for skin homeostasis. However, leaving excess sweat on the skin surface can induce itching in patients with AD. Several studies have shown that taking a shower after sweating is effective at relieving symptoms [75,76]. Therefore, if excess sweat remains on the skin surface, it can be washed away or wiped off [1].

## 8. Scratching Behavior

### 8.1. Scratching-Induced Aggravation of AD Lesions

Patients with AD often scratch their skin, resulting in further skin damage, which can lead to the exacerbation of eczematous lesions. It has been hypothesized that IL-33, IL-25, and TSLP, which are released from epidermal keratinocytes, act as endogenous “danger signals” or “alarmins” that alert the immune system to tissue damage [77]. It has been shown that the serum levels of IL-33 were higher in AD patients than in healthy individuals [78]. In addition, the patients’ serum IL-33 levels were correlated with their excoriation scores [78]. These findings suggest that tissue injuries caused by scratching of the skin might result in increased release of keratinocyte-derived cytokines, including IL-33, from damaged cells in patients with AD. Moreover, several previous studies have suggested that endogenous molecules that are released from tissues or cells by skin damage, including scratching, stimulate immune reactions in allergic dermatitis [79,80,81]. Therefore, scratching contributes to the exacerbation of eczematous lesions in AD.

### 8.2. Factors Influencing Scratching Behavior

Scratching can be caused by various itching-related and non-itching-related factors (Table 5). Itching signals are transmitted from the periphery to the brain via the dorsal horn by primary sensory neurons and spinothalamic tract neurons, and itching is induced by various mediators [77,82,83]. At the periphery, histamine acts as a pruritogen. Several type-2 cytokines, such as IL-4, IL-13, and IL-31, also directly activate sensory neurons [77,84,85,86,87]. Targeting the IL-31 pathway has been shown to be effective in patients with AD [84]. IL-4 and IL-13 are cytokines that are central to the pathogenesis of atopic disease and are primarily produced by Th2 cells [77]. Dupilumab is a human monoclonal antibody that is directed against the α-subunit of the IL-4 receptor. It blocks signaling from both IL-4 and IL-13 [85]. The administration of dupilumab resulted in significant improvements in inflammation and pruritus in AD patients [86]. Moreover, the epithelium-derived cytokine TSLP, which is deeply involved in the development of inflammatory responses in AD [77], acts directly on a subset of primary sensory neurons and induces itching [87].

In addition to these mediators, proteases (e.g., kallikreins, tryptase, endogenous/exogenous proteases) [89,90], neuropeptides (e.g., substance P) [91,92], and neurotrophic factors (nerve growth factor (NGF), artemin) [82,83,93] are considered to contribute to pruritus. Moreover, it has been reported that abnormal elongation of the sensory nerves into the epidermis occurs in the skin of AD patients [82]. In a mouse model of dry skin-induced itching, the expression of NGF was found to be elevated in the skin, and the number of intradermal nerve fibers increased [95]. On the other hand, semaphorins are a class of secreted membrane proteins, which function as axonal growth cone guidance molecules [82]. Semaphorin 3A is the first member of the semaphorin family that has been shown to cause growth cone collapse in neurons [96]. Semaphorin 3A regulates NGF-induced sprouting of sensory afferents in the spinal cord [97]. The abnormal elongation of the sensory nerves seen in AD is considered to be caused by an imbalance in the levels of nerve elongation factors, such as NGF, and nerve-repulsion factors, such as semaphorin 3A [82]. Such abnormal extension of the sensory nerves into the epidermis might lead to mechanical itching-induced dysesthesia, in which abnormal sensory states are induced by light cutaneous stimuli (alloknesis) [77,83]. In addition, patients with AD can experience strong itching in response to normal itch-evoking stimuli (hyperkinesis) [77,83]. Non-itching sensations, such as heat and pain, can also be experienced as itching by patients with AD [83].

The micro-opioid (beta-endorphin/micro-opioid receptor) and kappa-opioid (dynorphin A (DynA)/kappa-opioid receptor) systems are involved in the regulation of pruritus in the central nervous system. It has been reported that the micro-opioid and kappa-opioid systems are present in the human epidermis [98], suggesting that these systems might be responsible for peripheral pruritus. Kumagai et al. suggested that the micro-opioid system is itch-inducible, while the kappa-opioid system is itch-suppressive [99]. Interestingly, Tominaga et al. reported that the kappa-opioid system was downregulated in the epidermises of AD patients [100], indicating that patients with AD might experience itching due to the downregulation of the itch-suppressive system.

Patients with AD often scratch their skin in response to factors that are not related to itching. With regard to psychological aspects, it is known that the itching symptoms of AD patients can be worsened by stress and can be improved by stress management and behavioral modification [1]. Moreover, a vicious cycle of itching, scratching, and damaged skin might lead to scratching mainly out of habit instead of due to itching (habitual scratching). Thus, it is important to consider the possible involvement of such non-itching-related factors in addition to suppressing inflammation and pruritus in order to control the scratching behavior of AD patients.

## 9. Other Factors

### 9.1. Psychological Stressors

Emotional stress can increase not only itching but also the inflammation via the release of inflammatory mediators in AD patients [101,102]. The patients with AD had higher anxiety levels than healthy individuals, and those with a stronger trait anxiety than state anxiety showed elevated serum IgE levels and Th2 shifting [101]. Moreover, psychological variables affect serum levels of interferon-*γ* and IL-4 more in the patients with AD than healthy individuals [102]. These findings suggest that psychological stressors might affect the immunological reactions in the patients with AD.

### 9.2. Circadian Rhythms

Clinical symptoms in patients with allergic diseases including AD often depend on diurnal variations [103]. An intrinsic daily physiological rhythm called circadian rhythm is thought to affect the immune system. Several experimental studies have demonstrated that mutation in the circadian clock genes greatly affects immune responses [104,105,106]. Immune tolerance development is closely associated with the onset of immunological disorders. It has been shown that exposure to constant light impairs circadian rhythms, leading to the disturbance of tolerance induction [107]. Therefore, constant light environments such as lighting conditions at home in our night-active modern society may affect the development of AD.

## 10. Conclusions

Various aggravating factors, including both allergic and non-allergic factors, have been suggested to influence the course of AD. To identify factors that are specific to individual patients, it is important to consider whether the degree of eczematous lesions is affected by environmental changes, oral food challenges, or the avoidance of contact with suspected causative substances. In addition, we need to establish treatment strategies that take patients’ lifestyles into account. Furthermore, it is not necessary for AD patients to avoid sweating because it is important for skin homeostasis. However, leaving excess sweat on the skin can induce skin symptoms; therefore, sweat should be washed away or wiped off. Moreover, scratching leads to further skin damage, resulting in the exacerbation of eczematous lesions. It is important to consider clinical factors that might influencing scratching behavior (e.g., inflammation, stress, and habitual scratching). Future studies will contribute to the elucidation of the pathogenetic mechanism of AD, leading to improvements in the management of complicating factors.

## Figures and Tables

**Table 1:** Management of irritants.

Irritants	Management	References
Scrubbing the body	Wash the body gently without using nylon towels	[1,2]
Soap and shampoo	Use non-irritating soap and shampoo	[1,2,7]
Irritating clothes (e.g., wool-based clothes)	Choose suitable non-irritating clothes (e.g., cotton clothes)	[1,2,8,9]
Hair	Tie hair up	[1]
Saliva (during infancy)	Wash away or wipe off	[1]

**Table 2:** Representative allergens derived from *D. Pteronyssinus* and *D. farina* [16].

Allergen	Biochemical Name	Molecular Weight (kDa)
Der p 1/Der f 1	Cysteine protease	24 and 27
Der p 2/Der f 2	NPC2 family	15
Der p 3/Der f 3	Trypsin	31 and 29
Der p 4/Der f 4	Alpha-amylase	60 and 57.9
Der p 5/Der f 5	Unknown	14 and 15.5
Der p 6/Der f 6	Chymotrypsin	25
Der p 7/Der f 7	Bactericidal permeability-increasing like protein	26, 30, and 31
Der p 8/Der f 8	Glutathione S-transferase	27 and 32
Der p 9	Collagenolytic serine protease	29
Der p 10/Der f 10	Tropomyosin	36 and 37

**Table 3:** Evaluation and management of aeroallergens.

Allergen	Evaluation	Management	References
HDM	Serum-specific IgE antibody levels Skin prick testing and patch testing Evaluating changes in skin symptoms caused by environmental changes (e.g., trips, hospitalization, or moving house)	Ventilation Cleaning room Cleaning bedclothes with a vacuum cleaner, drying them in the sun, and washing sheets Encasing mattresses and bedding to protect patients from mites	[1,2,14]
Animal dander	Serum-specific IgE antibody levels Asking the patient about experiences involving the worsening of skin symptoms due to contact with animals	Giving up pets Washing pets Prohibiting pets in the bedroom	[1,2,14]
Pollen	Serum-specific IgE antibody levels Skin prick testing and patch testing Asking the patient about experiences involving the worsening of skin symptoms on exposed areas during a period of pollen scattering	Brushing pollen off clothes and washing face when arriving home Using protective glasses and masks Using air conditioning with pollen filters	[1,2,14]

**Table 4:** Representative contact allergens in AD patients.

Contact Allergens	Details of Contents	References
Metals	Nickel sulfate Cobalt chloride Potassium dichromate	[53,54,55]
Fragrances	Fragrance mix *Myroxylon pereirae* (Balsam of Peru)	[53,54,55]
Preservatives	Paraben mix Thiomersal	[53,54]
Rubber accelerators	Mercapto mix Thiuram mix Dithiocarbamate mix	[53,54]
Topical drugs	Steroids Antibiotics Moisturizer Eye drops	[53,54,55]
Cosmetics		[53]
Other chemicals	Lanolin	[53,55]

**Table 5:** Major factors influencing scratching in AD.

Factors	Details of Contents	References
Inflammatory mediators	Amines (histamine, serotonin)	[13,88]
	Cytokines (IL-4, IL-13, IL-31, IL-33, and TSLP)	[77,82,83]
	Proteases (kallikreins, tryptase, endogenous/exogenous proteases)	[89,90]
	Neuropeptides (substance P)	[91,92]
	Neurotrophic factors (nerve growth factor, artemin)	[82,93]
	Neurotransmitters (acetylcholine)	[94]
Environmental factors	Temperature, humidity, dry environments	[83]
Psychological stress		[1,83]
Habitual scratching		[1]
